# Deep brain stimulation in Parkinson’s disease: a comparison of accuracy and clinical outcomes of frame-based, frameless and frameless fiducial-less techniques

**DOI:** 10.1007/s10072-025-08102-0

**Published:** 2025-03-13

**Authors:** Riccardo Antonio Ricciuti, Matteo Maria Ottaviani, Fabrizio Mancini, Massimo Marano, Daniele Marruzzo, Francesca Barbieri, Riccardo Paracino, Pierfrancesco De Domenico, Serena Pagano, Vincenzo Di Lazzaro, Mauro Dobran

**Affiliations:** 1 Department of Neurosurgery, Azienda San Camillo Forlanini, Rome, Italy; 2 Department of Neurosurgery, Belcolle Hospital, Viterbo, Italy; 3 Department of Neurosurgery, Università Politecnica delle Marche, Ancona, Italy; 4 Department of Neurosurgery, Azienda Ospedaliera di Perugia, Perugia, Italy; 5 Research Unit of Neurology, Neurophysiology, Neurobiology and Psychiatry, Department of Medicine, Università Campus Bio-Medico, Rome, Italy; 6 Fondazione Policlinico Universitario Campus Bio-Medico di Rom, Rome, Italy; 7 Department of Neurosurgery and Gamma Knife Radiosurgery, IRCCS Ospedale San Raffale, Milan, Italy

**Keywords:** Deep brain stimulation, Parkinson’s disease, Subtalamic nucleus, Frame-based stereotactic surgery, Frameless stereotaxis

## Abstract

**Background:**

The effectiveness of deep brain stimulation (DBS) for Parkinson’s disease (PD) depends on implantation accuracy. DBS initially employed a stereotactic frame (frame-based, FB), but technological advancements led to the development of less invasive methods based on fiducial markers (F + F) or intraoperative imaging (F-F). This study compares the accuracy and efficacy of three DBS-STN implantation techniques.

**Methods:**

This retrospective study involved 18 patients with PD who underwent bilateral STN DBS between 2018 and 2023. Patients were divided into three groups: FB (*n* = 6), F + F (*n* = 7), and F-F (*n* = 5). Postoperative CT and preoperative MRI fusion were used to evaluate electrode accuracy via deviations from planned targets in x, y, z axes, and calculate the radial error (RE) and vector error (VE). We analyzed Unified Parkinson’s Disease Rating Scale (UPDRS) III scores in four “on/off medication-stimulation” combinations, LEDD, and disease stage before DBS, and 3 and 12 months post-DBS.

**Results:**

No statistically significant differences were observed between the three methods in|Δx| (FB = 1.30 ± 0.91; F + F = 1.05 ± 0.93; F-F = 1.33 ± 1.09 mm),|Δy| (FB = 0.95 ± 0.98; F + F = 1.11 ± 1.17; F-F = 1.28 ± 1.14 mm), RE (FB = 1.82 ± 0.29; F + F = 1,71 ± 0,36; F-F = 1,91 ± 1,49 mm) and VE (FB = 3,14 ± 0,35 mm; F + F = 4,92 ± 0,54 mm; F-F = 4,42 ± 1,22 mm). All groups demonstrated significant UPDRS III (> 50%) and LEDD reduction (> 40%) at 12 months, with no intergroup differences.

**Conclusions:**

The study concludes that all three techniques provide equivalent accuracy and clinical efficacy. Centers should select DBS-STN methods based on available resources and expertise.

## Introduction

Parkinson’s disease (PD) is a neurodegenerative disorder characterized by progressive motor symptoms that significantly impact daily life [1]. While pharmacotherapy remains the cornerstone of treatment, its long-term efficacy may wane, leading to motor fluctuations and side effects [2]. Deep brain stimulation (DBS) has emerged as a promising therapeutic option for alleviating symptoms and improving quality of life [3, 4]. DBS involves targeting brain regions like the Subthalamic Nucleus (STN), with precise electrode placement being critical to success. Misplaced electrodes account for 46% of treatment failures, often requiring revision surgery. A variety of techniques have been developed to guide the localization and implantation of DBS electrodes, with notable distinctions between frame-based methods, frameless methods, and fiducial-less methods [5, 6].

Frame-based (FB) methods have historically served as the gold standard for DBS surgery, employing stereotactic frames affixed to the patient’s skull to establish a stable reference system for precise electrode placement. Despite their reliability, FB methods may entail discomfort and inconvenience for patients due to the necessity of frame fixation [5, 7–9]. In contrast, frameless methods leverage advanced imaging modalities such as a combination of magnetic resonance imaging (MRI) and computed tomography (CT) scans in neuronavigational frameworks to guide electrode placement without the need for a stereotactic frame [10, 11]. Frameless DBS typically uses disposable, skull-mounted trajectory guidance in conjunction with neuronavigation and fiducial bone markers (F + F), which have been deemed essential for accuracy in frameless stereotactic surgery [5, 12]. However, frameless fiducial-less (F-F) methods have been developed to stream the DBS implantation procedure and reduce patients’ discomfort further. These techniques rely on intraoperative navigable imaging systems such as the O-Arm (Medtronic Inc.) and advanced neuronavigation to guide electrode placement without the need for fiducial markers or stereotactic frames [13, 14]. However, while offering greater patient comfort and flexibility, frameless methods may exhibit lower accuracy than frame-based approaches, primarily due to issues related to image distortion and registration errors [5]. Given the diversity of available techniques, a critical evaluation of their respective efficacy, accuracy, and procedural outcomes is paramount. This study reports our experience using three FB and frameless DBS techniques in PD patients to provide insights to optimize DBS strategies and clinical outcomes.

## Methods

### Patients

We retrospectively analyzed PD patients who underwent bilateral single STN-DBS using the FB, F + F, and F-F approaches between 2018 and 2023 at the Neurosurgery Department of Azienda Ospedaliera Universitaria delle Marche of Ancona (Italy), the Neurosurgery Department of Neurosurgery of Ospedale Belcolle of Viterbo (Italy) and at Fondazione Policlinico Universitario Campus Bio-Medico of Roma (Italy). Given that the number of tracks employed is a potential surrogate of accuracy, we selected only those patients who were implanted using single-track microelectrode recordings guidance.

### Surgical procedures

The procedures were completed in two stages on the same day: DBS electrode implantation on awake, sterilely draped patients for intraoperative assessments, followed by subcutaneous implantation of the internal pulse generator (Percept PC, Medtronic) in the left subclavicle under general anesthesia.

All patients underwent pre-operative brain MRI consisting of volumetric 3D T1 Gd-enhanced gradient echo sequences for trajectory planning and T2 turbo spin echo for STN targeting. For initial target localization, we employed the values suggested by Benabid et al. [15]—that is, 12 mm lateral, 2 mm posterior and 4 mm inferior to the mid-point of the AC (anterior commissure)–PC (posterior commissure) line acquired from the Schaltenbrand/Wahren atlas. These initial target coordinates were subsequently slightly changed to target the dorsolateral STN specifically visualized on the T2-weighted MR images. Accordingly, the AC–PC line and horizontal plan were used as references to calculate target coordinates in the x (laterality), y (anteroposterior) and z (cranio-caudality) axes. The coordinates (x, y, z) were expressed in millimeters relative to the mid-commissural point of the AC–PC line [16]. For all three techniques, a CT head scan was obtained before surgery and fused with the MRI study for STN targeting and trajectory planning. The trajectories were designed to avoid damaging the cortical veins, dural venous lakes, and lateral ventricles. In all cases, the first site of STN-DBS implantation was the left side, and all the operation times were registered.

#### Frame-based DBS

On the day of surgery, the CRW stereotactic frame^®^ (Radionics, Burling- ton, MA, USA) was fixed to patients’ skull and a CT scan was performed. Pre-operative MRI and CT scans were then fused using StealthMerge (Medtronic) and reformatted to produce images orthogonal to the AC–PC line and midsagittal plane. After confirming the accuracy of the MRI-CT fusion results, STN targeting and trajectory planning were performed as described earlier using the StealthStation™ S8 planning and navigation system (StealthStation S8, Medtronic). Patients were then positioned supine on the operating table and the frame was connected to a Mayfield fixation system (Integra, Cincinnati, OH, USA). Under local anesthesia, two frontal burr holes were drilled, and the dura mater opened on the left side to proceed with the intraoperative recording procedure.

#### Frameless DBS using bone fiducial markers

The day before surgery, seven bone fiducial markers were fixed on the skull under local anesthesia, followed by a fine-cut CT scan fused with pre-operative MRI (StealthMerge, Medtronic) for STN targeting and trajectory planning as above. On surgery day, patients were positioned supine, and a noninvasive reference frame (Head Tracker Frame, Medtronic) was secured to the forehead. Then, using a passive planar blunt probe, a non-sterile registration of the skull fiducial markers was performed to link images and surgical spaces. Burr hole entry points were marked, and patients were prepped sterilely. Surgery included bilateral skin incisions, burr holes, lead anchoring (Stimloc, Medtronic), and NexFrame© base placement. A second sterile registration (error < 0.4 mm) aligned the NexFrame© tower for trajectory planning using NexProbe and FrameLink software (Medtronic Inc.). The target depth was then calculated and set on the dedicated electrodes microguide (STar™ Drive; FHC, Inc), which was affixed to the NexFrame ring after dura opening and used to insert guide tubes and microelectrodes along the trajectory to begin the intraoperative recording procedure.

#### Frameless fiducial-less DBS

A preoperative volumetric CT scan was obtained and merged with the preoperative MRI for STN targeting and trajectory planning, performed as described earlier (StealthStation S8, Medtronic). This volumetric CT scan was employed as a reference imaging throughout the surgical procedure. On surgery day, patients were positioned supine, and a noninvasive reference frame (Head Tracker Frame, Medtronic) was secured to the forehead. A preliminary low-dose brain 3D CT scan was performed using an O-Arm system (O-arm O2, Medtronic, Inc., Minneapolis, MN, USA) to identify and mark the planned entry points over patients’ skin. After accurate sterile draping, the following surgical steps consisted in bilateral linear skin incision, realization of the two frontal burr holes, and placement of the lead anchoring device (Stimloc, Medtronic, Inc.) and the NexFrame© base around them. Subsequently, a high-definition 3D scan was obtained with the optic system solidly fixed to the skull serving as the foundation for definitive navigation and achieving a target registration error < 0.5 mm. The 3D scan was coregistered with other imaging data sets using the preoperative volumetric CT scan as reference imaging. The NexFrame© tower was then attached on the left side and aligned to the corresponding target through the planned trajectory using NexProbe© and FrameLink software (Medtronic Inc.). The target depth was then calculated and set on the dedicated electrodes microguide (NexDrive; Medtronic Inc.) affixed to the NexFrame© ring and used to insert guide tubes and microelectrodes along the trajectory. The dura mater was opened on the left side, and the intraoperative recording procedure started.

### Intraoperative recordings and macrostimulations

Single-track microelectrode recordings (MER, Medtronic Leadpoint) began 10 mm above the MRI-based target, advancing in 500 μm steps until no further STN activity was detected (typically 3 mm below the target). STN entry was marked by increased cellular discharge with irregular 25–45 Hz spikes, followed by quiet background noise and, if continued, high-frequency tonic firing from the substantia nigra. Depth and characteristics of STN activity were recorded. Channels with significant multi-unit activity over > 3 mm were selected for test stimulation. The microelectrode was replaced with a permanent quadripolar macroelectrode targeting the STN center. Macro-test stimulation (60s, 130 Hz) at various depths identified the channel with the largest therapeutic window for permanent implantation. The electrode was anchored with Stimloc and the dura closed with fibrin glue. The procedure was then repeated for the right side.

### DBS-electrode placement accuracy

A multi-slice CT scan was performed and fused with preoperative MRI to check the position of the implanted lead 30 days after surgery to avoid pneumocephalus. The final lead location was identified as the center of the beam-hardening artifact representing the deepest electrode contact as in [13, 14]. The x, y, and z AC-PC coordinates of this point were noted and differences with the planned lead AC-PC coordinates were calculated and expressed as both signed and absolute distance (mm) in each of the three axes (Δx, Δy, and Δz). These represent the directional component errors, with negative values indicating medial, posterior, and deep deviations. DBS accuracy was also assessed by calculating the radial error (RE) and the Euclidean error or vector error (VE). RE is the two-dimensional distance between the actual lead and the intended target and represents the error in the anteroposterior and mediolateral directions, reflecting mainly differences in the trajectories. It was measured on the axial plane of the intended DBS target and it was calculated as follows [5, 6, 10, 13, 17, 18]:

RE = √ (Δx^2^ + Δy^2^).

The Euclidean error or VE refers to the 3D distance between the target point and the tip of the first implant and it is calculated as follows:

VE = √ (Δx^2^ + Δy^2^ + Δz^2^).

The VE incorporates the z/depth error, which is less clinically relevant and is subject to more error when selecting the precise end of the implant as it is explored and modified intraoperatively according to microrecordings and clinical examination [5, 6, 10, 13, 14, 18, 19].

To compare implantation repeatability and precision, coefficients of variations (CV) were calculated on each direction (CV_x_, CV_y_, CV_z_) and an overall standard deviation (SD) for each group was computed as SD = √ (SD_x_^2^ + SD_y_^2^ + SD_z_^2^) [5], both for the left and right hemispheres.

### Clinical evaluations

Age at the time of DBS and years between PD onset and DBS were collected. For all patients drug scheduling was kept stable for more than six months before surgery and after DBS activation.

Variables that were assessed pre-operatively and post-operatively at 3 and 12 months included daily L-dopa dosage (LEDD), Hoehn and Yahr stage, L-dopa response, UPDRS IV and motor symptoms, which were evaluated using the Unified Parkinson’s Disease Rating Scale (UPDRS) part III in all the possible combination of stimulation On/Off and Medication On/Off [12, 20]. The L-dopa response was calculated as: [(UPDRS III_off med_ - UPDRS III_on med_)/ UPDRS III_off med_]*100, where UPDRS III scores are those pre-DBS. The change (%) in UPDRS III score comparing Off-stimulation state with On-stimulation state (in On-medication state) was considered the primary outcome to measure DBS motor improvement, since this measure is reflecting true DBS effectiveness:

$$\:DBS\:motor\:improvement\:$$

$$\eqalign{& \: = \frac{{\left( {UPDRS\:III\:score\:off\:stimulation} \right)\: - \left( {UPDRS\:III\:score\:on\:stimulaiton} \right)}}{{\left( {UPDRS\:III\:score\:off\:stimulation} \right)\:}} \cr & *100 \cr} $$

The secondary outcome measures of this study were the % change of DBS motor improvement and the % change in LEDD postoperative dosage compared with the preoperative one. These data were compared between the frame-based and frameless groups.

### Statistical analysis

Statistical analyses were conducted on clinical and surgery-related data (mean ± SD). Normality was assessed using the Shapiro–Wilk test. Group comparisons used the Mann-Whitney/unpaired t-test for paired data and one-way ANOVA/Kruskal-Wallis with Bonferroni’s post hoc for multiple comparisons. Within-group analyses evaluated surgical accuracy differences between left and right DBS implants (paired t-test/Wilcoxon) and clinical data changes over time (repeated-measure ANOVA/Friedman with Bonferroni’s post hoc). Statistical significance was set at *p* < 0.05.

## Results

### Subjects

A total of 18 PD patients were included in the study, 6 patients in the FB, 7 in the F + F and 5 in the F-F group. Patients’ average age at the time of STN-DBS was 53,8 ± 3,6 (range 48–57) for the FB group, 57,3 ± 4,2 for the F + F group (range 49–61), and 55,4 ± 4,3 for the F-F group (range 50–59). The mean length of disease duration for the FB group was 11,7 ± 1,4 (range 9–13), for the F + F group was 8,1 ± 1,7 (range 7–13), and for the F-F group was 8,8 ± 2,6 (range 6–13). The mean L-dopa responses were similar among groups, showing no statistically significant differences (FB = 51,47 ± 14,01%, F + F = 52,11 ± 14,95; F-F = 67,12 ± 7,90; *p* = 0,*31*).

### Accuracy evaluations

#### Absolute errors

Signed errors for each patient are reported in Table 1. Given the strong dependence of the final z coordinate from the microelectrode recordings performed during the procedure, we limited to report and analyze errors in the x and y axes. Accordingly, signed errors were used to estimate the mediality/laterality and anteriority/posteriority of the implanted DBS electrodes compared to the planned target coordinates. In the FB group we observed a medial placement of all electrodes compared to the planned target in 8/12 (67%) cases, 5 of which were in the left hemisphere. In the F + F group, 5/14 (35%) electrodes were placed medially, 3 of which in the left hemisphere, and 5/10 (50%) electrodes resulted medial to the planned target in the F-F group, 3 of which on the left side. Considering the y axis, the final localization of the electrodes resulted posterior to the planned target in 2/12 (16,7%) cases in the FB group (2/2 in the left hemisphere), in 5/14 (35,7%) cases in the F + F group (3/5 in the left hemisphere), and in 8/10 (80%) cases in the F-F group (4/8 in the left hemisphere).

**Table 1:** Signed errors in the X, y and Z axes for each patient in each of the three different DBS groups. Values are expressed in mm

	Frame-based			Frameless + Fiducilas				Frameless Fiducial-less			
	Δx	Δy	Δz		Δx	Δy	Δz		Δx	Δy	Δz
*P1 -Left*	-0,57	0	0,79	*P1 -Left*	-3,35	3,1	3,34	*P1 -Left*	-1,27	-0,8	2
*P1- Right*	1,08	0	2,29	*P1- Right*	0,98	0,45	5,66	*P1- Right*	0,23	-0,76	2,77
*P2 -Left*	1,14	0,11	2,49	*P2 -Left*	-0,94	0,19	2	*P2 -Left*	-1,89	-1,48	5
*P2 - Right*	-1,96	0,44	4	*P2 - Right*	-0,56	0,73	2	*P2 - Right*	2,25	-1,48	4,32
*P3 - Left*	-1,8	-1,35	4	*P3 - Left*	0,91	-0,15	6,52	*P3 - Left*	0	0	3,67
*P3 - Right*	-0,77	0,44	-2,53	*P3 - Right*	0,87	-3,54	6,52	*P3 - Right*	-0,82	0,08	3,62
*P4 - Left*	-1,53	2,99	1	*P4 - Left*	-1,11	0	-3	*P4 - Left*	-2,85	-3,27	4
*P4 - Right*	2,05	1,91	3	*P4 - Right*	-0,75	0,47	-3	*P4 - Right*	-0,06	-0,09	4
*P5 - Left*	-1,22	0,74	0	*P5 - Left*	2,35	-1,13	6,41	*P5 - Left*	1,07	-2,57	5
*P5 - Right*	0	2,32	0	*P5 - Right*	0	2	4	*P5 - Right*	-2,83	-2,3	3,62
*P6 - Left*	-3,32	-0,48	2,74	*P6 - Left*	0,21	-0,43	4,81				
*P6 - Right*	0,2	0,61	4	*P6 - Right*	-0,56	-1,17	6,49				
				*P7 - Left*	-3,35	3,1	3,34				
				*P7 - Right*	0,98	0,45	5,66				

Absolute errors were used to quantify the overall error independently of the mediality/laterality and anteriority/posteriority of the deviation. We found an overall absolute Δx error of 1,30 ± 0,91 mm for the FB group (CI 95% 0,79 − 1,82), 1,05 ± 0,93 mm for the F + F group (CI 95% 0,52 − 1,57), and 1,33 ± 1,09 mm for the F-F group (CI 95% 0,65 − 2,00), with no statistically significant differences among groups (*p* = 0,*74*). Significance was not reached either for differences observed in the y axis (*p* = 0,*77*), where the FB group showed the lowest mean absolute Δy of 0,95 ± 0,98 mm (CI 95% 0,39 − 1,50), the F + F group showed a mean absolute Δy of 1,11 ± 1,17 mm (CI 95% 0,45 − 1,77), and the F-F group presented the highest error of 1,28 ± 1,14 mm (CI 95% 0,57 − 1,99).

**Fig. 1:**
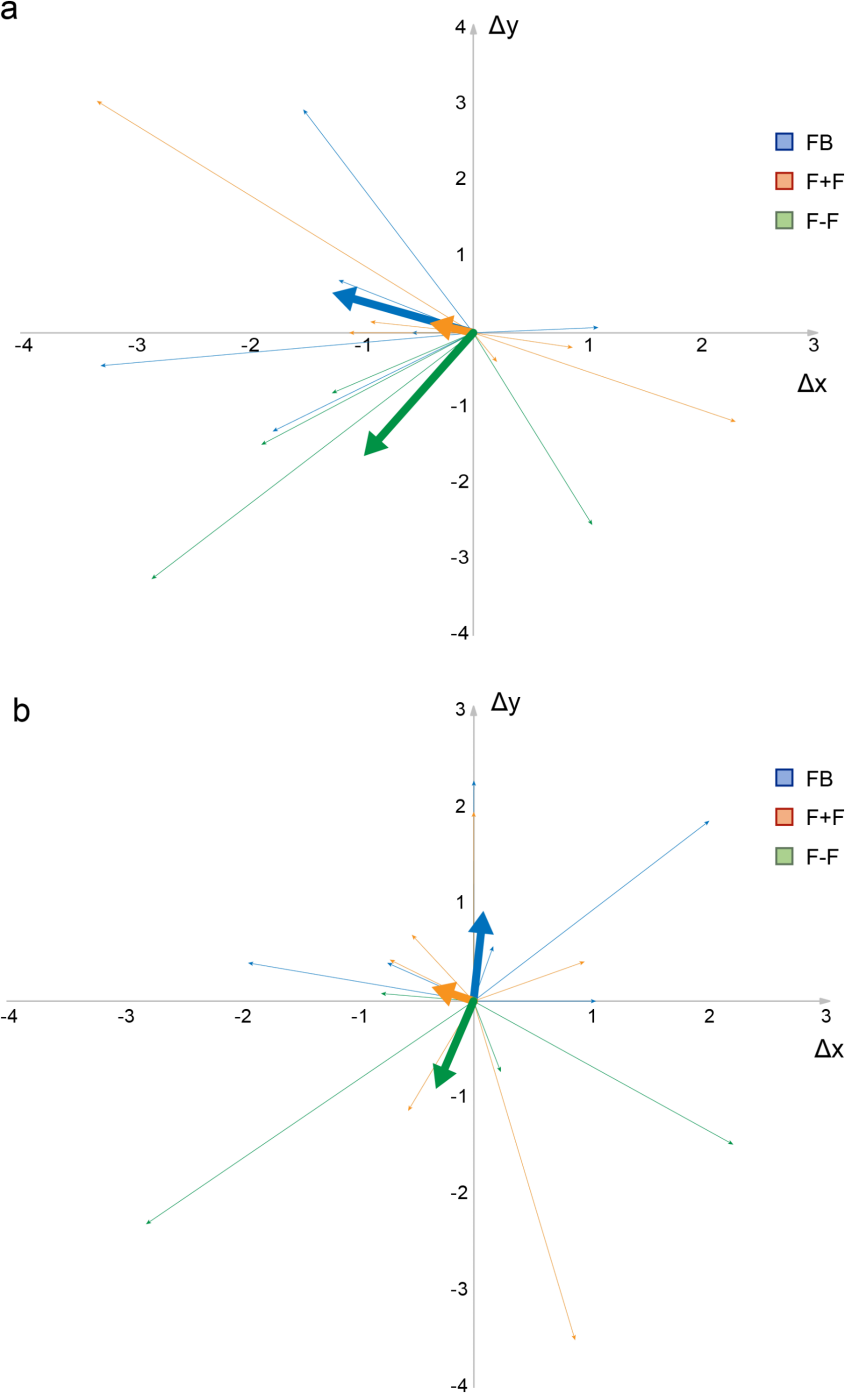
Graphical representation in the (x; y) plane of implanted electrodes coordinates in the left (**a**) and right (**b**) hemispheres for each group. Each electrode is represented via a thin vector that originates from the planned target (point 0;0) and terminates in the final electrode location related to the target (relative error). The thick vectors are the resulting vectors for each group and represents the overall tendency (mediality/laterality and anteriority/posteriority) for each specific DBS technique. Values are expressed in mm. FB: frame based; F + F: frameless with fiducials; F-F: frameless without fiducials

Considering electrodes implanted in the left hemisphere, the Δx error in the FB was 1,60 ± 0,94 mm (CI 95% 0,84 − 2,35), in the F + F group was 1,48 ± 1,15 mm (CI 95% 0,56 − 2,40), and in the F-F group was 1,42 ± 1,05 mm (CI 95% 0,49 − 2,34). In the right hemisphere, the Δx error in the FB group was 1,01 ± 0,86 mm (CI 95% 0,32 − 1,70), in the F + F group was 0,62 ± 0,34 mm (CI 95% 0,34 − 0,89), and in the F-F group was 1,24 ± 1,24 mm (CI 95% 0,15 − 2,32). No statistically significant differences were found either among groups (*p* = 0,*98* on the left side and *p* = 0,*5* on the right side) or comparing left vs. right implants within the same group (FB *p* = 0,*28;* F + F *p* = 0,*11;* F-F *p* = 0,*56*). Similarly, no statistically significant differences were found either for the y axis, both for intergroup (*p* = 0,*52* on the left side and *p* = 0,*7* on the right side) and intragroup comparison (FB *p* = 0,*98;* F + F *p* = 0,*43;* F-F *p* = 0,*37*). In particular, the mean Δy error for left implants was 0,95 ± 1,11 mm for the FB group (CI 95% 0,05 − 1,84), 0,83 ± 1,18 mm (CI 95% 0,11 − 1,77) for the F + F group, and 1,62 ± 1,32 mm (CI 95% 0,47 − 2,78) for the F-F group. The mean Δy error for right implants was 0,95 ± 0,93 mm for the FB group (CI 95% 0,21 − 1,70), 1,39 ± 1,2 mm (CI 95% 0,43 − 2,35) for the F + F group, and 0,94 ± 0,95 mm (CI 95% 0,11 − 1,78) for the F-F group. A graphical representation for all the electrodes implanted in the left and right hemispheres for each group is provided in Fig. 1.

CV and SD_tot_ are reported in Table 2. It can be observed that, with few exceptions, the CVs for the left implants were lower compared to right implants, suggesting higher repeatability of the first implant across the three different DBS techniques. Surprisingly, the F-F group showed the lowest CV values in all directions considered (x, y,z), while the F + F group the highest. This was confirmed also calculating the overall SD_tot_ as described before. Lastly, the repeatability in the z direction was higher compared to the x and y axes in all the three groups, possibly due influence of the MER to guide final position of the electrode in the z axis.

**Table 2:** Summary of coefficients of variation (CV) in the 3 directions for all the three groups and the relative overall standard deviation (SD) computed via combination of the SD in the three different directions

		Frame-based	Frameless + Fiducilas	Frameless Fiducial-less
**CV**_**x**_	*Bilateral*	2,74	8,77	2,67
	*Left implant*	1,21	6,08	1,57
	*Right implant*	13,99	228,75	7,47
**CV**_**y**_	*Bilateral*	1,89	38,09	0,92
	*Left implant*	4,4	5,56	0,81
	*Right implant*	0,98	10,94	1,09
**CV**_**z**_	*Bilateral*	1,11	0,99	0,24
	*Left implant*	0,81	1,07	0,31
	*Right implant*	1,44	1,02	0,16
**CV**_**tot**_	*Bilateral*	3,51	39,1	2,83
	*Left implant*	4,64	8,31	1,79
	*Right implant*	14,1	229,01	7,55
**SD**_**tot**_	*Bilateral*	2,81	4,09	2,22
	*Left implant*	2,56	4,32	2,38
	*Right implant*	3,08	4,22	2,17

#### Radial error

**Fig. 2:**
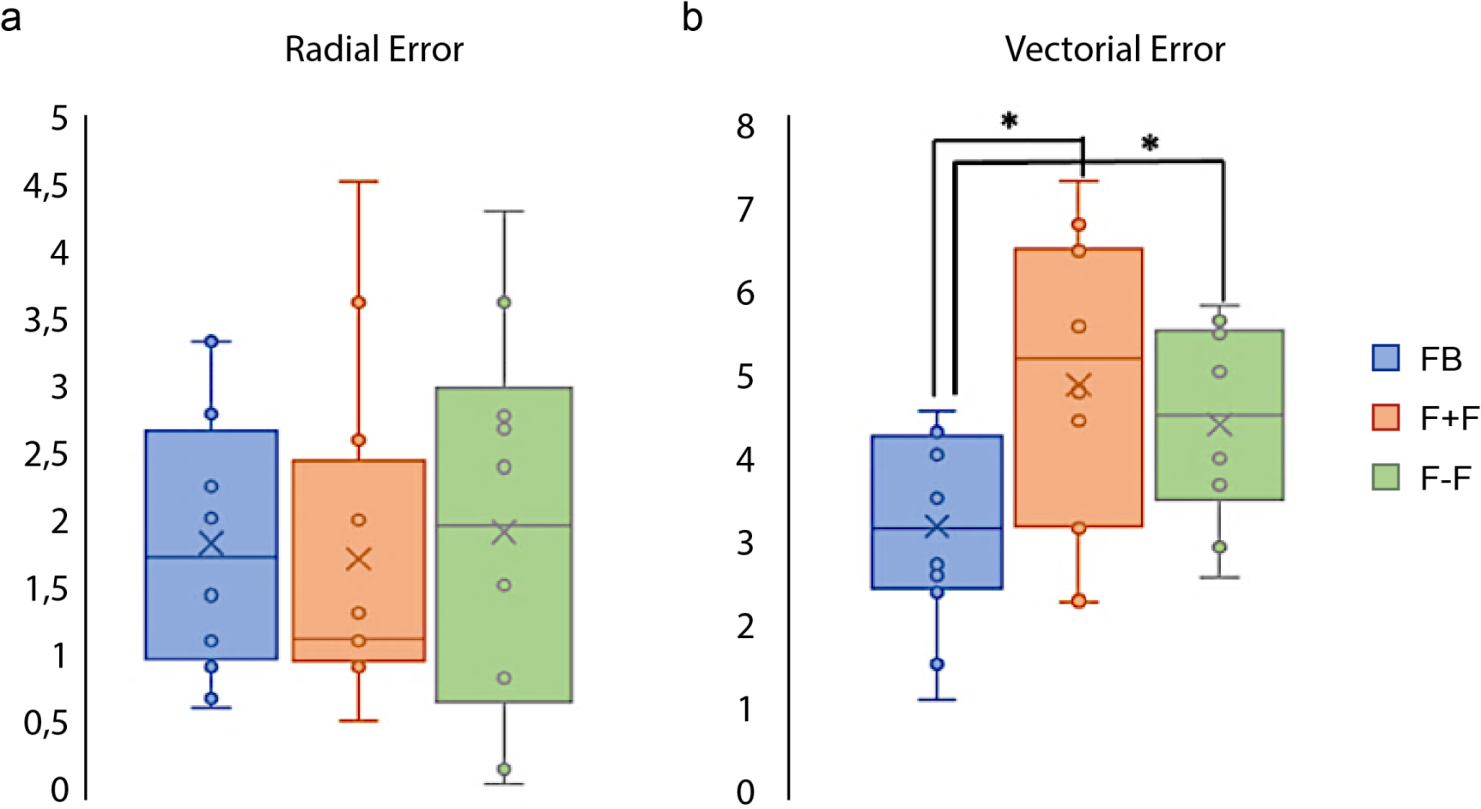
Box plots of Radial and Vectorial Errors for the three different DBS groups. Values are expressed in mm. FB: frame based; F + F: frameless with fiducials; F-F: frameless without fiducials. *p < 0,05

The mean bilateral RE was 1,82 ± 1,00 mm for the FB group (CI 95% 1,25 − 2,39), 1,71 ± 1,27 mm for the F + F group (CI 95% 0,98 − 2,42), and 1,91 ± 1,49 mm for the F-F group (CI 95% 0,98 − 2,83), without statistically significant differences among groups (*p* = 0,*93*). The mean REs for the left implant were 2,03 ± 1,17 mm (CI 95% 1,08 − 2,95), 1,77 ± 1,55 mm (CI 95% 0,53 − 3,01), and 2,20 ± 1,63 mm (CI 95% 0,79 − 3,61) for the FB, F + F and F-F groups, respectively. The mean REs for the right implant were 1,62 ± 0,87 mm (CI 95% 0,92 − 2,32), 1,64 ± 1,07 mm (CI 95% 0,79 − 2,49), and 1,61 ± 1,49 mm (CI 95% 0,31 − 2,92) for the FB, F + F and F-F groups respectively. No statistically significant differences were found either among the three groups (*p* = 0,*92* for the left side and *p* = 0,*67* for the right side) and comparing left vs. right side within the same group (FB *p* = 0,*52*; F + F *p* = 0,*86*; F-F *p* = 0,*51*). Results are summarized in Table 3 and graphically represented in Fig. 2.

**Table 3:** Mean absolute errors in the X, y and Z axes, mean radial error (RE), and vectorial error (VE) for bilateral, left and right implants. Values are reported as mean ± standard deviation and expressed in mm

		Frame-based	Frameless + Fiducilas	Frameless Fiducial-less
**|Δx|**	*Bilateral*	1,30 ± 0,91	1,05 ± 0,93	1,33 ± 1,09
	*Left implant*	1,60 ± 0,94	1,48 ± 1,15	1,42 ± 1,05
	*Right implant*	1,01 ± 0,86	0,62 ± 0,34	1,24 ± 1,24
**|Δy|**	*Bilateral*	0,95 ± 0,98	1,11 ± 1,17	1,28 ± 1,14
	*Left implant*	0,95 ± 1,11	0,83 ± 1,18	1,62 ± 1,32
	*Right implant*	0,95 ± 0,93	1,39 ± 1,2	0,94 ± 0,95
**|Δz|**	*Bilateral*	2,24 ± 1,48	4,48 ± 1,80	3,8 ± 0,92
	*Left implant*	1,83 ± 1,49	4,34 ± 1,87	3,93 ± 1,23
	*Right implant*	2,63 ± 1,48	4,61 ± 1,9	3,67 ± 0,58
**RE**	*Bilateral*	1,82 ± 1,00	1,71 ± 1,27	1,91 ± 1,49
	*Left implant*	2,03 ± 1,17	1,77 ± 1,55	2,20 ± 1,63
	*Right implant*	1,62 ± 0,87	1,64 ± 1,07	1,61 ± 1,49
**VE**	*Bilateral*	3,14 ± 1,21	4,92 ± 1,87	4,42 ± 1,22
	*Left implant*	2,93 ± 1,49	4,9 ± 1,87	4,67 ± 1,51
	*Right implant*	3,36 ± 0,95	4,94 ± 2,04	4,16 ± 0,96

#### Vectorial error

**Fig. 3:**
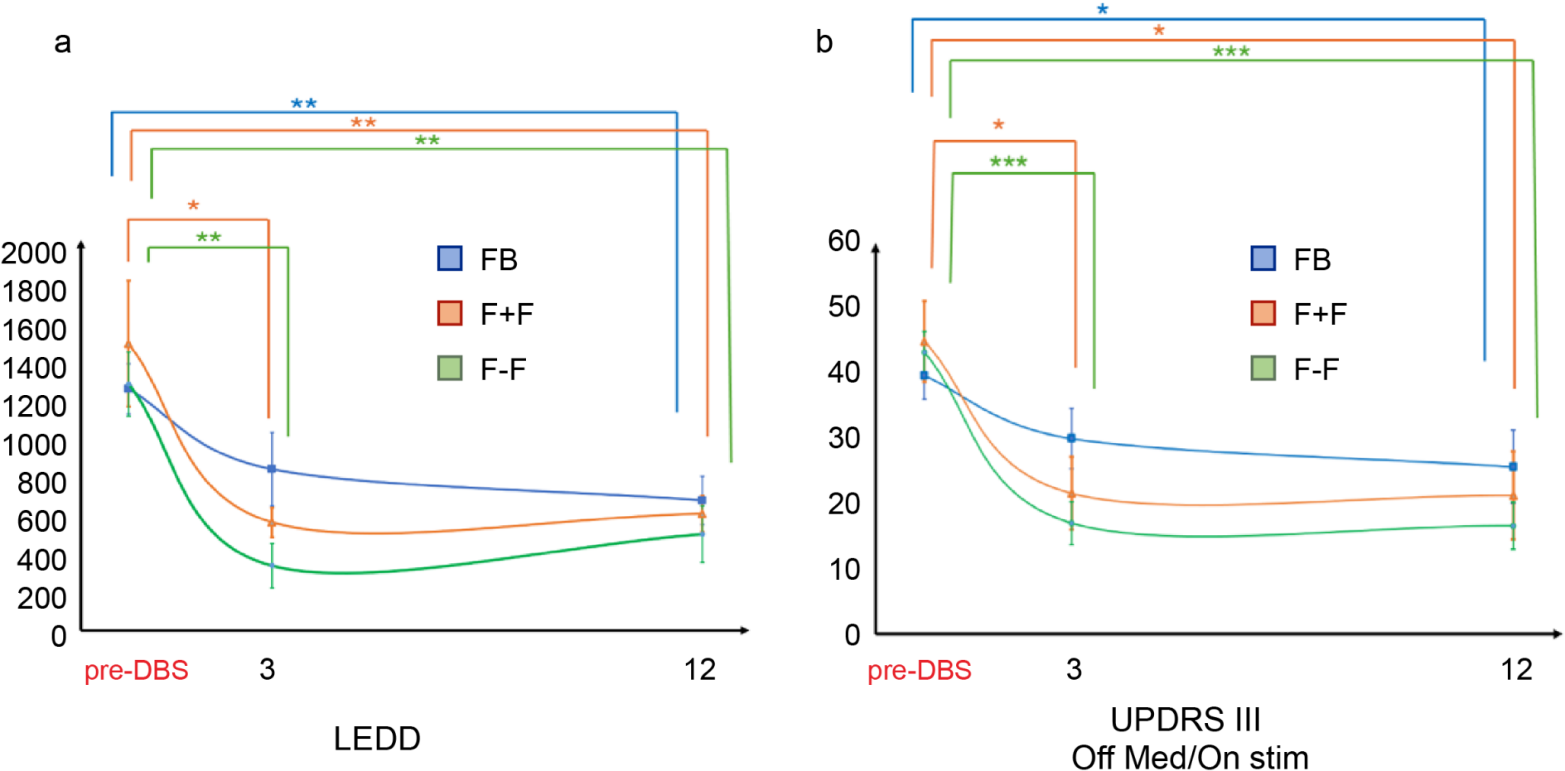
(**a**) Levodopa equivalent daily-dose and (**b**) UPDRS III Off med/On stim values pre-DBS, 3- and 12-months post-DBS in the three groups. FB: frame based; F + F: frameless with fiducials; F-F: frameless without fiducials. *p < 0,05, **p < 0,01

The mean bilateral VE differed significantly among groups (*p* = 0,*019*). In particular, the FB group showed a significantly lower VE = 3,14 ± 1,21 mm (CI 95% 2,46 − 3,83) compared to both the F + F group having a mean VE = 4,92 ± 1,87 mm (CI 95% 3,86 − 5,98), and the F-F group with a VE = 4,42 ± 1,22 mm (CI 95% 3,66 − 5,17) (*p* = 0,*01* and *p* = 0,*02* respectively). The mean VEs for the left implants were 2,93 ± 1,49 mm (CI 95% 1,73 − 4,12), 4,90 ± 1,87 mm (CI 95% 3,40 − 6,40), and 4,67 ± 1,51 mm (CI 95% 3,35 − 5,98) for the FB, F + F and F-F groups, respectively. The mean VEs for the right implant were 3,36 ± 0,95 mm (CI 95% 2,60 − 4,12), 4,94 ± 2,04 mm (CI 95% 3,31 − 6,58), and 4,16 ± 0,96 mm (CI 95% 3,32 − 5,01) for the FB, F + F and F-F groups respectively. No statistically significant differences were found either among the three groups for both the left and right implants (*p* = 0,*13* and *p* = 0,*2* respectively) and comparing left vs. right side within the same group (FB *p* = 0,*56*; F + F p = *0*,*97*; F-F *p* = 0,*54*). Results are summarized in Table 3 and graphically represented in Fig. 3.

### Clinical results

Clinical data are summarized in Table 4. First, we observed that DBS caused a significant reduction of the LEDD at 12 months post-DBS in all groups. In particular, LEDD values decreased from 1237,2 ± 311,7 to 665,5 ± 302,7 in the FB group (*p* = 0,*009*), from 1467,6 ± 851,2 to 597,9 ± 244,4 in the F + F group (*p* = 0,*02*) and from 1261,0 ± 366,0 to 493,0 ± 322,5 in the F-F group (*p* = 0,*007*). Moreover, a significant reduction was achieved also at 3 months compared to the pre-DBS LEDD in the F + F (*p* = 0,*02*) and F-F groups (*p* = 0,*001*) (Table 4). No statistically significant differences were observed among the three groups for the pre-DBS, 3-months and 12-months post-DBS LEDD values (shown in Fig. 3).

The mean Hoehn and Yahr score in Off medication was higher in the FB group compared to the other groups (Table 4) at the pre-DBS evaluation (*p* = 0,*001* vs. F + F and *p* = 0,*0001* vs. F-F), at 3 months (*p* = 0,*012* vs. F + F and *p* = 0,*02* vs. F-F) and at 12 months post-DBS *p* = 0,*012* vs. F + F and *p* = 0,*02* vs. F-F). However, no statistically significant intragroup differences were observed at each time interval in all groups, both in Off and On conditions (Table 4).

Motor evaluations were performed via calculation of the UPDRS III score in all possible combinations (On med/On stim; Off med/On stim; On med/Off stim; Off med/ Off stim) at pre-DBS, 3-months, and 12-months post-DBS (Fig. 3). Intergroups analysis did not show any statistically significant difference comparing the same condition at each time point (*p* = 0,*2*). The DBS motor improvements at 3 and 12 months were the lowest for the FB group (30,86 ± 16,57% and 50,62 ± 21,96%, respectively) and the highest for the F-F group (62,27 ± 13,03% and 63,54 ± 14,33%, respectively). The only statistically significant difference was found when comparing the DBS motor improvement between the FB and the F-F group at 3 months (*p* = 0,*007*).

The FB group showed the lowest pre-DBS UPDRS IV score but without statistically significant differences with the other groups (*p* = 0,*83* vs. F + F and *p* = 0,*26* vs. F-F). Similarly, the UPDRS IV scores at 3 and 12 months did not differ among groups (*p* = 0,*11* and *p* = 0,*13* respectively). However, we observed a statistically significant improvement from the pre-DBS UPDRS IV score to the one at 3 months and at 12 months in all groups (*p* = 0,*0018* and *p* = 0,*001* for the FB group; *p* = 0,*024* and *p* = 0,*022* for the F + F group; *p* = 0,*002* and *p* = 0,*001* for the F-F group).

**Table 4:** Summary of all clinical data collected for each group at each time point (pre-DBS, 3- and 12-months post-DBS). FB: frame based; F + F: frameless with fiducials; F-F: frameless without fiducials. *p < 0,05 - **p < 0,01 - ***p < 0,001 compared to the pre-DBS condition

			FB	F + F	F-F
**LEDD**	*Pre-DBS*		1237,2 ± 311,7	1467,6 ± 851,2	1261,0 ± 366,0
	*3 months*		825,5 ± 458,2	553,6 ± 199,7*	331,6 ± 251,2*
	*12 months*		665,5 ± 302,7**	597,9 ± 244,4*	493,0 ± 322,5**
**Hoehn and Yahr stage**	*Pre-DBS*	Off med	4 ± 0	2,9 ± 0,6	2,8 ± 0,2
		On med	2,3 ± 1,5	2 ± 0	2 ± 0
	*3 months*	Off med	4 ± 0,63	2,91 ± 0,58	2,83 ± 0,29
		On med	3 ± 0,42	2 ± 0	2 ± 0
	*12 months*	Off med	4 ± 0,63	2,91 ± 0,58	2,83 ± 0,29
		On med	3 ± 0,42	2,16 ± 0,41	2,16 ± 0,29
**UPDRS III**	*Pre-DBS*	On med	19,33 ± 6,68	20,83 ± 4,62	18,67 ± 3,06
		Off med	39,67 ± 8,89	44,86 ± 16,39	43,20 ± 7,12
	*3 months*	Off med/ Off stim	43,67 ± 12,50	44,86 ± 16,39	43,2 ± 7,12
		Off med/On stim	30,04 ± 11,29	21,57 ± 14,80	17,00 ± 7,38
		On med/Off stim	15,66 ± 6,65	19,33 ± 6,98	14,75 ± 8,22
		On med/On stim	19,33 ± 15,23	12,83 ± 7,36	12,25 ± 9,60
	*12 months*	Off med/ Off stim	50,17 ± 11,65	47,00 ± 17,50	43,60 ± 7,23
		Off med/On stim	25,67 ± 13,82	21,29 ± 17,77	16,6 ± 8,05
		On med/Off stim	21,17 ± 9,87	21,5 ± 9,85	15,00 ± 8,52
		On med/On stim	20,67 ± 16,54	14 ± 9,53	13,00 ± 9,63
**UPDRS IV**	*Pre-DBS*		8,83 ± 2,64	9,14 ± 2,47	10,80 ± 2,86
	*3 months*		2,83 ± 2,31**	4,14 ± 4,48*	3,2 ± 2,28**
	*12 months*		2,17 ± 2,4**	4,00 ± 3,10*	2,80 ± 1,92**
**Motor improvement**	*Pre-DBS ◊ 3 months*		30,86 ± 16,57	51,33 ± 32,56	62,27 ± 13,03
	*Pre-DBS ◊ 12 months*		50,62 ± 21,96	56,99 ± 33,02	63,54 ± 14,33

## Discussion

Our study showed that FB, F + F, and F-F STN-DBS had equivalent spatial accuracy, which aligns with results reported in other series [5, 6, 11]. No significant differences were found in absolute errors on the x- and y-axes, though the FB method differed on the z-axis compared to F + F and F-F, with no differences between the frameless groups. However, z-axis differences are influenced by intraoperative MER adjustments, which could be reflected in greater repeatability observed in the z direction compared to the x and y axes in all the three groups in terms of CV and SD. Moreover, the z coordinate is usually the most difficult to measure postoperatively because it depends on the manual choice of the end of the electrode tip artifact on the post-operative CT scan. Finally, the error on the z-axis can be mitigated by choosing different stimulating contacts on the DBS electrode. Accordingly, RE values were similar across methods, while VE was lower for the FB method. Both RE and VE were within reported ranges [8, 10, 13].

We observed no significant brain shift effects between sides but, albeit not statistically significant, higher accuracy for right implants and lower CVs for left implants, indicating higher repeatability for first implants. We evaluated directional errors to determine if inherent system inaccuracies might cause deviations in electrode positioning on individual planes. We focused our analysis on the x and y axes as planned coordinates on these axes are those that contribute the most to the definition of the trajectories for leads placement. Directional errors revealed a relatively homogeneous probability of medial or lateral deviation, while relative errors in the y-axis revealed a prevalent tendency for anterior placement in the FB group and a higher probability of posterior placement for the F + F and the F-F groups. Although there were no significant differences in the length of errors (in mm), the considerably higher percentage of posterior deviation in the F-F group raises the possibility that methodological or sample size factors might be contributing to these findings. Indeed, the small number of cases—particularly in subgroup analyses for the left hemisphere—could have amplified variability and influenced the statistical interpretation of these directional errors.

PD patient profiles were homogeneous, with no significant differences in clinical characteristics or motor improvement at 3 and 12 months. Accuracy within 1–2 mm did not affect motor response or side effects, aligning with existing literature [5, 6].

Our study demonstrated comparable accuracy and clinical outcomes among the three DBS methods used by the same group. Balancing the advantages and disadvantages of each approach, we suggest calibrating the choice according to single center resources and experience, but larger studies are needed to support this. Limitations of the current study include the small sample size and discrepancies with previous meta-analytic results underscore the need for cautious interpretation. Balancing the advantages and disadvantages of each approach, we suggest calibrating the choice according to single center resources and experience, but future studies with larger cohorts and standardized measurement protocols are warranted.

## Data Availability

All data generated or analyzed during this study are included in this article. Further enquiries can be directed to the corresponding author.
